# Assessing regional economic growth through green financial policy: Insights from PSM-DID model on 107 cities of China

**DOI:** 10.1016/j.heliyon.2023.e19568

**Published:** 2023-09-01

**Authors:** Cheng Hu, Yan Wang

**Affiliations:** a School of Economics and Management, Hanjiang Normal University, Shiyan, Hubei 442000, China; b Dept. of Economy and Trade, Hubei Industrial Polytechnic, Shiyan, Hubei 442000, China

**Keywords:** Green financial reform and innovation pilot zone, Regional economy, Industrial structure

## Abstract

The crucial role of green finance policies in advocating environmental sustainability cannot be overlooked and has resulted in an increased attention in recent years. Despite their significance, the connection between these policies and economic growth remains disputed among policymakers. This study presents an empirical analysis that aims to uncover the relationship between green finance policies and economic growth rates by analysing the data from 107 cities in China, spanning a period of 16 years from 2003 to 2019. The research focuses on the implications of green finance reforms and innovation pilot zones by employing PSM-DID model to evaluate their effects on the regional GDP by segregating the industries in primary, secondary, and tertiary industries. The results indicate that the green finance reforms and innovation pilot zones are associated with reduced growth rates of regional GDP for secondary industries. However, the growth rates of the tertiary industry are positively affected. Thus, the study offers valuable guidance and recommendations to policymakers and cities seeking to join the pilot zones for green financial reform to foster economic growth while supporting environmental sustainability. The findings of the study also contribute to the understanding of how targeted policy interventions, such as the establishment of pilot zones, can influence economic dynamics at the regional level and describes the complex relationship between policy interventions, economic growth, and industry composition, particularly in the context of environmental sustainability.

## 1 Introduction

**Fig. 1:**
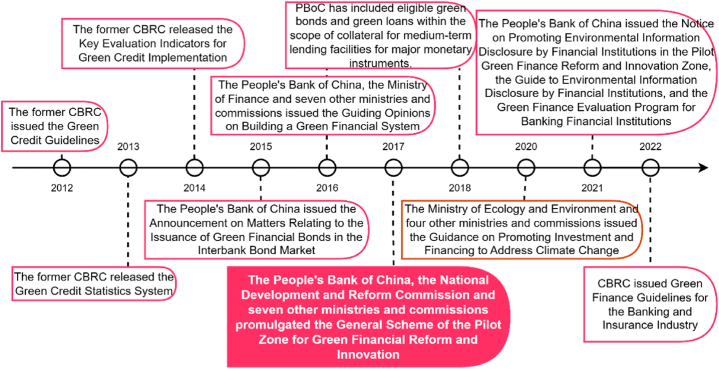
Timeline of China's green finance policy development in the past decade.

Green finance is a field where practice comes first and then a theoretical system is formulated. While comparing the development of green finance in developed countries in the West and China, we find that their development models differ significantly. In the developed countries of the West, represented by the United States and the United Kingdom, a variety of financial institutions and non-governmental organizations are the main participants in green finance, which is mainly based on market mechanisms. Government involvement only occurs when green finance has reached a certain level of development, resulting in a “top-down” development model that is influenced by market demand. It took longer for green finance to take hold in developing countries, represented by China, and the initial creation of the green financial system primarily relied upon government power and was supported by government departments and regulators, resulting in a government-oriented “top-down” approach. There has been an improvement in China's green finance policy system over the past decade, and the author compares and contrasts the timeline of green finance policy development in China (Fig. 1).

The relevance of green finance in promoting sustainable economic growth has become a subject of academic inquiry, leading to varying conclusions among scholars. For instance, Ref. [1] research examined the impact of green finance development on business activities and national economic growth in ASEAN countries from 2008 to 2019 using the indicators of central banks. The study found that green finance, including green investment, green security, and green credit, positively correlate with economic development in ASEAN countries. In contrast, Ref. [2] study created a comprehensive index of green finance, including green credit, green insurance, green securities, and carbon finance. They concluded that the relationship between green finance and economic growth in China is weak, and green finance cannot effectively link industrial restructuring, environmental protection, and economic growth. While these papers used micro financial instruments as explanatory variables for verification, this research diverges from the literature, which examines the impact of green finance policy using indicators. This paper used quasi-natural experiments to evaluate the impact of green finance policy on regional economic growth. A few scholars have utilized the green finance reform pilot area to conduct empirical research, but their focus is different from this study as they have not verified the impact of the policy on the regional economy. References [3] study focuses on the impact of the green finance reform pilot zone policy on urban green development, concluding that green finance can promote urban green innovation and reduce energy consumption as urbanization significantly contributes to carbon emissions [4]. Similarly, Ref. [5] investigation highlights that green financial reform pilot policies can significantly propel regional green technological innovation. Thus, these studies offer valuable guidance and insights for policymakers seeking to promote economic growth through green finance while supporting environmental sustainability. The aim of this research was to investigate how the implementation of the green financial reform and innovation pilot cities, together with the large cities identified in the seventh census of China, impacted the regional economic development from an economic efficiency perspective. In order to achieve this, a multi-temporal double difference model (DID) was developed, which allowed for the assessment of changes in regional national income growth rates over the period 2003–2019.

The results highlighted a significant and noteworthy suppressive effect of the green financial reform pilot policy on regional national income growth rates. This finding was assessed through a robustness test, which further supported the validity and reliability of the findings. Overall, these outcomes could have implications for policymakers and practitioners alike, as they suggest that the implementation of green financial reform policies may have broader economic consequences within the Chinese context. Moreover, the following study provides a basis for further research to investigate and explore these findings in greater depth, to better understand the underlying mechanisms and implications for future policy making. Also, the study revealed that the primary and secondary industries were significantly suppressed, whereas the tertiary industry was significantly boosted. In light of this finding, cities with diverse industry types may use it as a reference for making policy decisions. As well, the report provides theoretical support and empirical evidence for whether they should choose to apply to become green financial reform test sites. We believe that cities with a predominantly tertiary industry profile should actively seek to become pilot areas for green financial reform.

This paper comprises of three essential contributions. Firstly, it investigates the impact of establishing a pilot zone for green financial reform on regional economic growth and industrial structure growth, thereby adding to the empirical research on green pilot zones and green finance. Secondly, the use of a quasi-natural experiment approach through the exogenous event of establishing a pilot zone minimizes endogenous issues, which strengthens the estimation results and provides reliable empirical evidence concerning the connection between green financial reform and regional economic development. Thirdly, this research employs a proximity test that scrutinizes the impact of green finance reform pilot programs on similar cities, accounting for the heterogeneity and significant differences that exist amongst cities. This approach enhances our understanding of the scope and implications of green financial reform policies and their effectiveness in promoting sustainable economic growth. Overall, these findings contribute significantly to the literature on green finance, pilot zones, and the broader environmental and economic policies.

The remainder of this paper proceeds as follows: Section 2 offers a comprehensive review of the relevant literature in the field, providing a detailed background for the study. Section 3 outlines the data sources and models used to analyze the economic efficiency implications of the green financial reform pilot cities and large cities from a macro-policy perspective. In Section 4, the empirical results and robustness tests are presented, which allow for the assessment of the effectiveness of the green financial reform policy. Section 5 then delves into a discussion of the underlying reasons for the empirical results obtained, offering insights into the potential factors and mechanisms at play. Finally, Section 6 concludes the paper with a summary of the key findings, implications, and recommendations for policy-makers and practitioners involved in green finance and regional economic development. By following this structured approach, the study provides a thorough analysis of the impact of green financial reform policies on economic development in China, contributing to the broader literature on environmental and economic policies.

## 2 Literature review

### 2.1 Definition of green finance

In the past few years, in-depth discussions have taken place between international organizations and governments regarding the role that green finance plays in policymaking at the global level. These discussion reveal that the concept of green finance is understood differently by different institutions. As defined by some, green finance involves investments and financing activities that are related to the environment [[6], [7], [8]] and theseactivities must be related to the environment, eco-friendly industries, or sustainable development. There is an emphasis on the importance of green finance in promoting the flow of funds to projects that can improve environmental quality, mitigate climate change, and adapt to it. It also applies to activities that improve the protection and exploitation of natural resources. As stated by other institutions, green finance is a financial service policy that aims to minimize the impact on the environment [9,10]. It is believed that its purpose is the transition to a sustainable global economy to achieve economic growth. One of the aims of the green financing is the transition from the traditional fossil fuels to renewable sources of energy and it had been investigated that the transition of industry reliance from fossil fuels to renewable energy, have a negative effect on per-capita carbon emissions [11]. While achieving this transition, pollution and greenhouse gas emissions are reduced, waste is minimized, and natural resources are used more efficiently. The question arises: What are the consequences of green finance for the environment and the economy?

### 2.2 Environmental impact of green finance

The above definitions of green finance may differ. However, there is consensus on the concept that green finance is the financing of economic projects that are beneficial to the environment. Green finance has been proven to be effective in promoting environmental upgradation from various perspectives by scholars. On the basis of micro-enterprises, green investment is considered a successful financial strategy that positively impacts enterprise performance [12,13], thus indirectly improving environmental quality. As well as being able to impact the performance of enterprises, green financial policy will also play the role of a regulatory constraint on companies. By implementing environmentally friendly laws and regulations, green finance can assist high-polluting businesses in transforming themselves into environmentally friendly businesses. Thus, improving the quality of the environment and reducing emissions [14,15]. At a macro level, the world is actively exploring measures to mitigate, climate change. Numerous studies conducted in recent years, including those by Refs. [[16], [17], [18], [19]]; and [20]; have provided substantial evidence that green financial policies exert a positive impact on the environment. It is also pertinent to note that some scholars have pointed that some green energy policies like renewable energy can have negative consequences for Moreover, Ref. [21] have gone on to showcase the effectiveness of green finance in reducing the ecological footprint and transforming the environmental landscape in Asia using the original least squares baseline model. This is a significant breakthrough, as it suggests that implementing green finance policies could have a tangible impact on the environment in various regions across the globe. In addition, Ref. [22] have leveraged G7 data and advanced panel approaches to gauge the potential of green investments and financing in considerably reducing carbon dioxide emissions. In order to reduce the environmental impact [23] recommend that renewable energy has mitigating effect on the carbon emission while they revisit the Environmental Kuznets Curve (EKC) hypothesis in a global context by considering 208 economies from 1990 to 2018. Furthermore, green energy not only contributes to the environment but it also adds in the economic growth [24]. This study holds immense value as it could pave the way for incorporating green investments and financing as potent tools for reducing carbon emissions and combating climate change. Overall, these findings underscore the pivotal role of green financial policies in promoting sustainable development and ensuring a greener future.

### 2.3 The impact of green finance on regional economy

In the introduction to this paper, it is stated that different researchers have come up with different methods and different verification results for determining whether green finance contributes to economic development or not. The most common method of assessing the role of green finance in promoting economic growth [25,26], as well as supporting the upgrading of industrial structures [[27], [28], [29]], is to use green credit, green bonds, green insurance, and other financial products as explanatory variables. By using these explanatory variables, it is possible to verify that green finance is capable of promoting economic growth and supporting industrial structure upgrading in order to produce a green economy. The economic growth can reduce income inequality in the region which has also been identified as a contributing factor in the environment [23]. Among them, several have confirmed the impacts of green finance development on the industrial structure by using the Generalized Method of Moments (GMM) to construct a model based on the Dynamic Panel System [[30], [31], [32]]. In terms of selecting variables to explain, the focus is primarily on the environment [33], the energy economy [34], or green development [35]. For example, when taking environmental quality into account as the explained variable in this study, it is believed that there is a positive correlation between green finance and environmental quality, while there is a negative correlation between environmental quality and economic growth.

Most of the existing studies on the green finance domain focus on analyzing the impact of green financial products, such as green credit, green investment, and green financing, on the environment, enterprise development, and industrial structure. However, there are only a few studies that are conducted from the perspective of green financial policies. Furthermore, a considerable amount of literature on green financial reform pilot zones centers around the role of corporate and regional green innovation, with little attention paid to the pilot effect of green financial reform pilot zones on regional economies and industries. Therefore, this paper's main objective is to examine the effects of green financial reform and innovation pilot zones on regional economic growth and industrial structure through the Propensity Score Matching (PSM) and Difference-in-Differences (DID) modeling techniques. By doing so, this study aims to bridge the gap in the literature and provide a more comprehensive understanding of the efficacy of green financial policies in promoting sustainable economic growth and restructuring regional industries.

## 3 Data and methods

### 3.1 Sample selection and data sources

The State Council approved the first batch of green financial reform and innovation pilot zones in June 2017. Among them are Huzhou and Quzhou in Zhejiang Province, Guangzhou City in Guangdong Province, Ganjiang New Area in Jiangxi Province, Gui'an New Area in Guizhou Province, and Karamay City, Hami City, and Changji Prefecture in Xinjiang Uygur Autonomous Region, a total of eight places in five provinces. This marks the beginning of a new phase for China's green finance, combining a top-down, regional exploratory approach with a top-down, top-level design approach. As of November 2019, Lanzhou New Area in Gansu Province was approved as the country's ninth pilot zone for green financial reform and innovation. This is the only pilot zone for green financial reform and innovation in the Yellow River Basin. The Pilot Zone for Green Financial Reform and Innovation in Chongqing was officially launched in August 2022.

This study suggests the use of green reform pilot zones as a natural experiment to evaluate their effect on regional economic growth. The treatment group for this experiment comprises ten cities with green financial reform pilot zones, while cities without green financial reform pilot zones make up the control group. The double difference method is utilized to measure and compare the impact of green reform and innovation pilot zones on economic growth in regions across these two groups. This approach helps in isolating the effects of green financial policies on the economy by controlling for any pre-existing differences between the treatment and control groups. By using this method, the study aims to provide a more comprehensive understanding of the effectiveness of green financial reform policies in promoting sustainable economic development.

In order to meet the prerequisites for using the double difference method, the following pre-processing was performed in selecting the sample in this paper:

(1) Except for Quzhou, Huzhou, Guangzhou, Karamay, Changji, Karamay and Chongqing City, not all pilot areas are divided by cities in the pilot area documents. This paper contains a pilot reform area of ten cities. How were they selected? Nanchang City is used as the sample because Jiangxi Ganjiang New Area is located on the bank of Ganjiang River in the north of Nanchang City. The Gui'an New Area is located at the junction of Guiyang City and Anshun City, so Guiyang City and Anshun City are used as samples. The Lanzhou New Area is located north of Lanzhou City in Gansu Province, which is why Lanzhou City was used as a sample. This paper does not include Chongqing in its list of reform pilot zone cities since it was approved in August 2022.
(2) Considering the fact that the industrial structure of big cities is complete, it is necessary to select big cities to ensure the comparability between the treatment group and the control group. According to the “2020 China Population Census County-by-County Data” published by the Office of the Seventh National Population Census Leading Group of the State Council on October 22, there are 105 big cities. Of the 105 big cities identified, four are county-level cities and only have a single industrial structure, so that should be eliminated. A total of 107 cities' sample data has been collected after adding 10 cities in the pilot area and subtracting the cities that overlap with the big cities (107 = 105-4 + 10-4).
(3) To minimize the influence of external factors such as the COVID-19 pandemic, the research period for this study was selected from 2003 to 2019. The source of the data is the China Urban Statistical Yearbook and the CSMAR database collected over this period, and any missing data are interpolated to ensure completeness. Therefore, a comprehensive set of balanced panel data was compiled and covers 107 major cities in China over the period from 2003 to 2019. By utilizing this data, the study aims to provide a reliable and accurate assessment of the effects of green financial reform pilot zones on economic growth in different regions of China.

### 3.2 Model

In this paper, green financial policy is used as the explanatory factor and regional economic growth is used as the explained factor, where green financial policy is measured by green financial reform pilot zones and regional economic growth is measured by the growth rate of regional GDP and the share of value added of each industry in GDP. The impact effects of pilot zones on the local economy are identified by comparing the differences in the impact effects of cities before and after they become pilot zones (experimental group) and cities that are not pilot zones (control group). The double difference model constructed is as follows.

(1) $$gr_{it}=\alpha +\alpha_{i}+\alpha_{t}+\beta *did_{it}+\gamma *controls_{it}+\varepsilon$$

(2) $$g{kr}_{it}=\alpha +\alpha_{i}+\alpha_{t}+\beta *did_{it}+\gamma *controls_{it}+\varepsilon$$

where did denotes the policy effect, gr is the gross regional product, gkr is the percentage of each industry as a share of GDP (k = 1, 2, 3), and controls are the matrix of control variables, as detailed in Tables 1 and 2.

**Table 1:** Definition and description of explained variables.

Type	Name of explained variable symbol	Symbol	Mean (%)	Std. Dev. (%)
Regional Economic Growth	GDP growth rate	gr	11.19802	4.057145
Industrial Structure Growth	Primary Industry as Percentage to GDP	g1r	8.998477	3.626137
	Secondary Industry as Percentage to GDP	g2r	48.47606	5.761404
	Tertiary Industry as Percentage to GDP	g3r	42.50464	6.653495

**Table 2:** Descriptive statistics of control variables.

Type	Symbol	Definition & Unit	Mean	Std. Dev.
Macroeconomic data	pe	Local General public budget expenditure (ten thousand yuan)	4565029.94	4515465.14
	consume	Total retail sales of consumer goods (ten thousand yuan)	13080698.50	10560816.71
	investfa	Total investment in fixed assets (ten thousand yuan)	17440409.72	11705398.25
Industrial structure by employee data	pg2r	The ratio of employees in the secondary industry (%)	48.76277	6.989383
	tel	The total count of individuals working in the industries of information transmission, computer services, and software development (ten thousand people)	1.988473	3.539245
Data conducive to the development of green financial policies	fin	The number of employees in the financial sector (ten thousand people)	3.231878	2.759025
	env	The number of employees in the water, environment and public facilities management industry (ten thousand people)	1.241452	0.730179
	lgca	Green coverage in built-up areas (%)	39.47658	3.567599

The following study employs Propensity Score Matching (PSM) combined with a Difference-in-Differences (DID) model which is advantageous for several reasons. PSM aids addressing the problem of selection bias in observational studies as in non-experimental settings, treatment assignment is not random, and there may be systematic differences between the treatment and control groups does this by balancing the covariates between the treatment and control groups, which helps to reduce bias in the estimates. PSM also provides more accurate estimates of the causal effect of the treatment on the outcome variable as it balances the distribution of observed covariates between the treated and control groups. Since our study combines PSM with DID model so it strengthens the causal inference as it compares the pre and post treatment outcomes allowing for the estimation of causal effects and DID model further isolates the treatment effect from other factors that may affect the outcome over time.

### 3.3 Variable selection

#### 3.3.1 Explained variable

The primary objective of this study is to examine the impact of green financial reform pilot zones on the domestic economy of China. In doing so, the growth rates of gross regional product (gr), the proportion of the value added of the primary industry to GDP (g1r), the proportion of the value added of the secondary industry to GDP (g2r), and the proportion of the value added of the tertiary industry to GDP (g3r) are utilized as explanatory variables. By analyzing these variables, the study aims to explore the influence of the green financial reform pilot zones on both overall economic growth and industrial development in different regions of China. The findings of the study are presented in Table 1, where the impact of the test regions on the regional economy is examined in detail.

#### 3.3.2 Explanatory variables

The explanatory variable of this study is whether it is a green financial reform pilot area. Double differential variables are primarily used to measure it. The did statistic is calculated as the product-term of the dummy variables in the processing period and the dummy variables in the processing group, that is, did = dummy variables in the processing period * dummy variables in the processing group. As long as it is officially approved, this year and later will be valued at 1, while other years will be valued at 0. The value of dids is zero in cities that are not approved as pilot areas.

#### 3.3.3 Control variables

To ensure the reliability and robustness of the findings, an array of control variables (Table 2) have been selected in this study. The first category includes macroeconomic indicators that are positively correlated with the level of economic development in each city. These indicators comprise public finance expenditures (pe), retail sales of consumer goods (consume) and investments in fixed assets (investfa). The second category, based on employee data, assesses the development of the industrial structure in each city. The ratio of employees in the secondary industry (pg2r) and the number of employees in the information transmission computer service and software industry (tel) have been selected in this category. As per conventional wisdom, a high secondary industry employment ratio indicates that the city is economically dependent on secondary industries. Similarly, the level of IT talent in a city is considered to be indicative of tertiary industry growth. Finally, the third category comprises data that is supportive of the development of green financial policies, including the number of employees in the financial sector (fin), water, environment and public facilities management industry (env), as well as the extent of green coverage in built-up areas (lgca). These factors reflect the effective promotion and implementation of green financial policies. Based on these considerations, eight control variables were selected for the study.

## 4 Empirical results

### 4.1 Baseline regression results

Table 3 reports the results of the baseline model regression. As a result of not including control variables, the coefficient of impact of the establishment of the pilot green financial reform and innovation zone on the GDP growth rate of the area is −1.122, which is highly negative at the level of 1%. Resulting in a reduction in regional economic growth as a result of green financial reform. The impact coefficient on the proportion of the primary industry is −0.522, which is significantly negative. It indicates that the pilot policy has a restraining effect on the development of the primary industry. The impact coefficient on the proportion of the secondary industry is −0.894, which is also significantly negative, demonstrating that the pilot policy also plays a restraining role in the development of the secondary industry. The coefficient of influence on the growth rate of the tertiary industry is 1.059, which is strongly positive at the level of 1%. This implies that the pilot policy can promote the development of the urban tertiary industry.

**Table 3:** Baseline regression results.

	(1)	(2)	(3)	(4)	(5)	(6)	(7)	(8)
	sgr	sgr	sag1r	sag1r	sag2r	sag2r	sag3r	sag3r
did	−1.122***	−0.222	−0.522***	−0.322**	−0.894***	0.074	1.059***	0.109
	(0.201)	(0.145)	(0.179)	(0.135)	(0.188)	(0.138)	(0.189)	(0.125)
sape		−0.142**		0.219***		0.040		−0.150***
		(0.056)		(0.052)		(0.053)		(0.048)
saconsume		−0.715***		−0.484***		−0.613***		0.794***
		(0.049)		(0.045)		(0.046)		(0.042)
sainvestfa		−0.091***		−0.048**		−0.022		0.047**
		(0.025)		(0.023)		(0.024)		(0.022)
sapg2r		0.129***		−0.366***		0.537***		−0.265***
		(0.021)		(0.020)		(0.020)		(0.018)
satel		0.002		0.024		−0.077*		0.053
		(0.041)		(0.038)		(0.039)		(0.036)
safin		0.279***		−0.112**		0.086		−0.017
		(0.060)		(0.055)		(0.057)		(0.051)
saenv		0.286***		−0.123***		0.133***		−0.049
		(0.042)		(0.039)		(0.040)		(0.036)
salgca		−0.286***		−0.089***		−0.043**		0.088***
		(0.019)		(0.018)		(0.018)		(0.017)
_cons	0.017	0.003	0.008	0.005	0.014	−0.001	−0.016	−0.002
	(0.023)	(0.016)	(0.020)	(0.015)	(0.021)	(0.015)	(0.022)	(0.014)
*N*	1819	1819	1819	1819	1819	1819	1819	1819
*Time fixed*	Yes	Yes	Yes	Yes	Yes	Yes	Yes	Yes
*Individual fixed*	Yes	Yes	Yes	Yes	Yes	Yes	Yes	Yes

Standard errors in parentheses.

*, **, and *** indicate that the statistical value is significant at the 10%, 5%, and 1% levels, respectively.

### 4.2 Robustness testing

#### 4.2.1 Changing the measure of explanatory variables

In order to further verify the results of the initial analysis, a different approach was employed to handle the time variable. Given that the establishment of the first green financial reform pilot zone occurred in the midyear of 2017, assigning a value of 1 for that year may exaggerate the policy impact on the test area. To account for this concern, the time variable was adjusted to 0.5 for the year of establishment, while the values for the remaining years were assigned by following the baseline regression equation. The regression results after the adjustment are presented in Table 4, which indicates the effects of green finance on regional economic growth, secondary industry growth, and tertiary industry growth. The regression coefficients remained statistically significant, and positive/negative effects on different indicators persisted at varying confidence levels. These findings are consistent with the results of the baseline regression tests presented in Table 3, and add to the evidentiary support for the conclusion that green financial reform pilot zones play a significant role in promoting economic development and restructuring industries in different regions of China. The robustness of the analysis is thus reaffirmed by these supplementary findings.

**Table 4:** Outcome of regression analysis with altered explanatory variable measurement.

	(1)	(2)	(3)	(4)	(5)	(6)	(7)	(8)
	sgr	sgr	sag1r	sag1r	sag2r	sag2r	sag3r	sag3r
did	−1.231***	−0.213	−0.598***	−0.352**	−0.951***	0.164	1.151***	0.047
	(0.229)	(0.165)	(0.204)	(0.153)	(0.214)	(0.157)	(0.216)	(0.142)
sape		−0.143**		0.218***		0.039		−0.148***
		(0.056)		(0.052)		(0.053)		(0.048)
saconsume		−0.716***		−0.484***		−0.613***		0.794***
		(0.049)		(0.045)		(0.046)		(0.042)
sainvestfa		−0.091***		−0.048**		−0.023		0.047**
		(0.025)		(0.023)		(0.024)		(0.022)
sapg2r		0.129***		−0.366***		0.538***		−0.266***
		(0.021)		(0.020)		(0.020)		(0.018)
satel		0.001		0.023		−0.078**		0.055
		(0.041)		(0.038)		(0.039)		(0.036)
safin		0.280***		−0.111**		0.088		−0.018
		(0.060)		(0.055)		(0.057)		(0.051)
saenv		0.287***		−0.123***		0.135***		−0.050
		(0.042)		(0.039)		(0.040)		(0.036)
salgca		−0.286***		−0.090***		−0.043**		0.088***
		(0.019)		(0.018)		(0.018)		(0.017)
_cons	0.016	0.003	0.008	0.005	0.012	−0.002	−0.015	−0.001
	(0.023)	(0.016)	(0.020)	(0.015)	(0.021)	(0.015)	(0.022)	(0.014)
*N*	1819	1819	1819	1819	1819	1819	1819	1819
*Time fixed*	yes	Yes	Yes	Yes	Yes	Yes	Yes	Yes
*Individual fixed*	yes	Yes	Yes	Yes	Yes	Yes	Yes	Yes

Standard errors in parentheses.

*, **, and *** indicate that the statistical value is significant at the 10%, 5%, and 1% levels, respectively.

#### 4.2.2 PSM-DID test

**Fig. 2:**
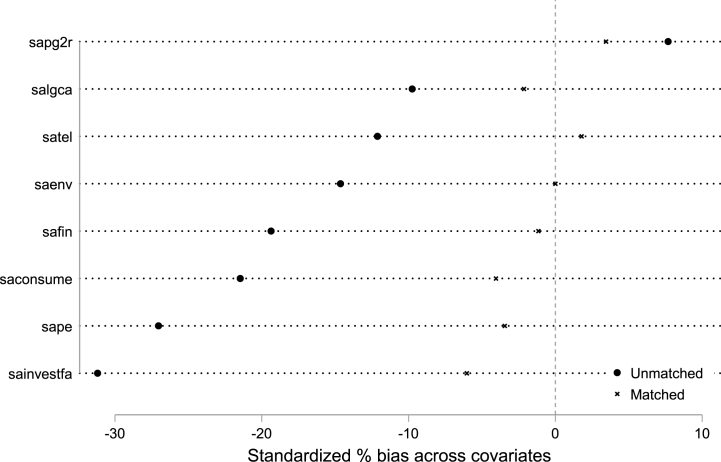
Kernel matching chart.

Since the cities selected as pilot zones are not chosen at random, they may be selected because these pilot cities have certain characteristics. Moreover, policies are generally implemented first in pilot demonstration areas, which may already have a fairly high level of economic development. Therefore, in order to further ensure the validity of the regression results and eliminate the influence of the characteristic factors of the pilot cities on the empirical results, it is necessary to find cities similar to the pilot cities in the non-pilot cities for comparison tests. Fig. 2 shows that the results of kernel matching are within the interval (−10%, 10%) on the horizontal axis, which means that matching is successful and the subsequent DID regression can be performed. Furthermore, the results of nearest neighbor matching with a caliper range of 0.00780668 also demonstrate that most indicators fall within the (−10%, 10%) interval.

After conducting PSM-DID, Table 5 presents the regression findings which demonstrate that the regional economic growth rate, secondary industry, and tertiary industry yielded consistent results as the baseline regression. Notably, the once insignificant primary industry now exhibits significant negative outcomes at a 5% level, as indicated by the kernel matching model. This suggests that green finance impedes the advancement of primary industries.

**Table 5:** Regression results after PSM-DID.

	(1)	(2)	(3)	(4)	(5)	(6)	(7)	(8)
	newsgr	newsgr	newsag1r	newsag1r	newsag2r	newsag2r	newsag3r	newsag3r
did	−11.822***	−11.571***	−5.215***	−5.236***	−7.815***	−7.389***	9.622***	9.262***
	(1.420)	(1.502)	(1.533)	(1.571)	(2.493)	(2.319)	(1.759)	(1.612)
sape		0.433		0.317		0.408		−0.517
		(0.409)		(0.244)		(0.325)		(0.324)
saconsume		−0.469*		−0.363		−0.751***		0.845***
		(0.237)		(0.236)		(0.275)		(0.300)
sainvestfa		0.100		−0.001		0.165		−0.141
		(0.106)		(0.065)		(0.134)		(0.112)
sapg2r		−0.006		−0.243*		0.320*		−0.145
		(0.087)		(0.127)		(0.177)		(0.128)
satel		0.128		0.026		−0.003		−0.017
		(0.154)		(0.166)		(0.144)		(0.168)
safin		−0.076		0.104		0.173		−0.211
		(0.402)		(0.337)		(0.360)		(0.352)
saenv		−0.150		−0.303		−0.204		0.346
		(0.206)		(0.265)		(0.296)		(0.360)
salgca		−0.396**		−0.086		−0.001		0.047
		(0.200)		(0.075)		(0.082)		(0.071)
_cons	0.286***	0.283***	0.060**	0.059**	0.156***	0.144***	−0.168***	−0.156***
	(0.022)	(0.024)	(0.024)	(0.025)	(0.039)	(0.035)	(0.028)	(0.026)
*N*	1790	1790	1790	1790	1790	1790	1790	1790
*Time fixed*	Yes	Yes	Yes	Yes	Yes	Yes	Yes	Yes
*Individual fixed*	Yes	Yes	Yes	Yes	Yes	Yes	Yes	Yes

Standard errors in parentheses.

*, **, and *** indicate that the statistical value is significant at the 10%, 5%, and 1% levels, respectively.

#### 4.2.3 Assessing the parallel trend assumption in the analysis

**Fig. 3:**
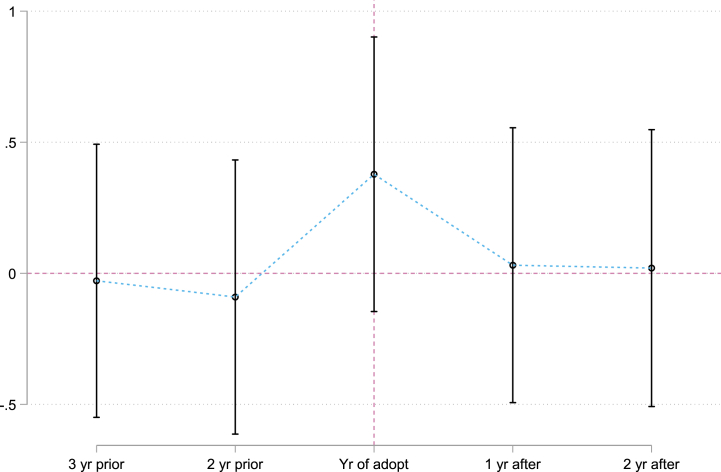
The results of parallel trend test through visual representation.

To ensure that the DID model is reliable and can be utilized for research purposes, it is imperative to acknowledge that the treatment and control groups conform to the parallel trend assumption. This assumption implies that in the absence of treatment, the difference in outcomes between the two groups is relatively constant over time. By satisfying this basic requirement, it is possible to confidently attribute any observed differences in outcome after the introduction of the treatment to the actual impact of the intervention under consideration. Thus, we carefully evaluated whether the parallel trend assumption holds before attempting to draw conclusions from the DID model results. Using 2014 as a hypothetical year for cities with pilots established in 2017, and so on, the pilots were constructed 3 years before, 2 years before, current, one year after, and two years after the policy was implemented. Fig. 3 shows that the equilibrium trend assumptions are satisfied for the two target variables, pilot and non-pilot cities, before the policy occurs, indicating that the regression is valid.

#### 4.2.4 The use of placebos

**Fig. 4:**
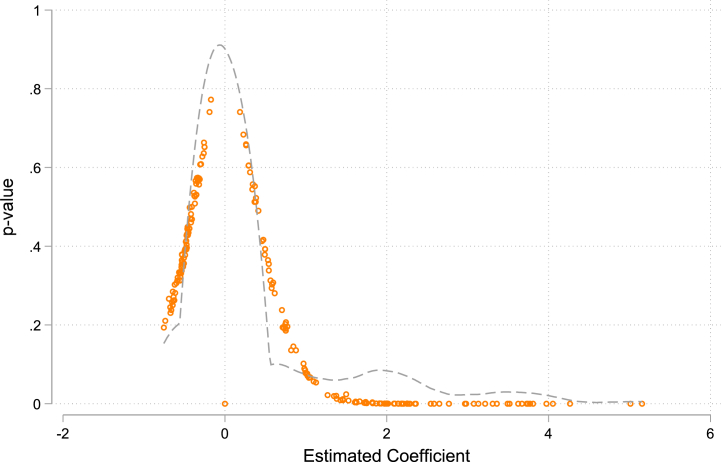
Placebo test result.

To ensure that the results obtained from the implementation of the Green Financial Reform and Innovation Test Area are free from the influence of unidentified factors, a placebo test was conducted. This study utilized data from 500 samples taken from 107 cities, with only the first period number (2003) retained for each ID. To match the previous data, a random selection of 10 cities was made from each sampling. The matched cities were then used as a dummy treatment group in the regression, while the remaining cities were considered the control group. The scatter plot in Fig. 4 indicated that the regression results were normally distributed. The cities selected at random did not reflect the effects of the test area, leading to the conclusion that the findings of this paper successfully passed the placebo test.

## 5 Discussion

The above empirical results could be attributed to a variety of factors. First and foremost, the coefficient of the impact of the green reform pilot policy on regional economic growth is extremely negative, indicating that the implementation of the green financial reform pilot policy will significantly decrease the regional economy's growth rate. This may be due to the fact that under the constraint of the environmental development goal, green financial development may crowd out other financial resources. In other words, environmental requirements have been raised, and investments that previously yielded high returns but did not meet environmental standards are no longer considered viable. As a result, GDP growth slows down and shifts into green, sustainable growth, instead of the crude growth of the past.

It is also evident from the second result that there is a restriction on the development of primary and secondary industries. Possibly, this is due to differences in the energy demand structure among different types of industries. Green financial policies may help to promote the transformation of old into new sources of energy. As energy and industrial structure contributes to the ecological footprint of an economy [36]. Consequently, the traditional “high pollution, low output” primary and secondary industries are abandoned by capital and do not receive adequate funding. In this way, a higher level of industrial structure will be achieved.

The hypothesis mentioned earlier has been corroborated by its impact on the tertiary industry. The regression analysis performed indicates a noticeable and positive influence of the tertiary sector's growth. Based on these observations, it can be deduced that the adoption of green financial policies seems to be conducive to the expansion and progress of the tertiary industry. Furthermore, the implementation of the green finance reform and innovation policy could aide in the transformation of primary and secondary industries into tertiary ones. While there may be implications from this shift, its potential benefits could lead to greater diversification and sustainability in the long run.

The application process for becoming a green financial reform pilot zone is underway in many cities. However, based on the empirical results of this study, green financial reform pilot zones will inhibit regional economic growth. Thus, not all cities are eligible to participate in the pilot program. Aside from that, different cities have different industrial structures as well as different characteristics of their industrial pillars. It is therefore necessary to determine whether to pursue green financial policy support in conjunction with their own development objectives.

## 6 Conclusion and policy recommendations

In China, ten pilot zones for green financial reform and innovation have been approved across seven provinces, autonomous regions, and municipalities directly under control of the central government. Furthermore, a growing number of cities are seeking to join these designated pilot zones. Against this backdrop, this paper seeks to evaluate whether such pilot zones have the potential to stimulate regional economic growth. To achieve this, the study employs quasi-natural experiments across 107 Chinese cities from 2003 to 2019. By examining the data from these cities, the research aims to assess the relationship between the implementation of pilot zones and their potential impacts on economic growth in the region.

Based on the results we deduce that the development of both primary and secondary industries may be restricted, whereas the tertiary sector seems to have gained a significant momentum to grow. These findings held constant after a set of robustness tests were executed, which lends credibility to the empirical link between green financial reform pilot zones and short-term regional economic growth. What makes this discovery noteworthy is that it provides robust and reliable empirical evidence that local authorities can reference when considering whether cities should apply to become pilot zones. Furthermore, these conclusions could inform policy decisions related to the potential advantages and drawbacks of prioritizing the green finance sector over other industrial sectors within a region.

Based on the findings of our study we suggest that the central government should designate green financial reform pilot zone based on the industrial structural and characteristics of the cities; those cities with a strong tertiary industry should be given priority for pilot zone designation as our findings suggest that tertiary industry benefits more from the establishment of these pilot zones. The growth momentum in the cities with primary focus on tertiary industries can be further leveraged by giving substantial support for green financial reform and innovation in those cities. The impact of green finance policies should not be viewed as directly promoting economic growth, as their primary goal is to support sustainable and low-carbon development. Therefore, not all cities may be suitable as green reform pilot zones, since it depends on their individual characteristics. Local authorities should exercise caution while implementing green finance policies in cities with primary or secondary industry dominance as the following study suggests that these industries may experience short-term constraints on their economic growth due to the impact of green finance policies. Therefore, policymakers should cautiously evaluate the possible adverse effects and consider alternate strategies to support sustainable development in these industries. Furthermore, it is also important to consider contextual considerations such as the designations and functional positioning of each pilot zone should be tailored to the unique characteristics, resources, industrial structures, economic sizes, and financial development bases of the respective cities as our findings reveal that the effect of pilot zones on the development across industries is subjective to the level at which an industry is operating.

However, cities with a primary focus on the primary or secondary industry may be negatively impacted by green finance policies, which could pose a short-term constraint on their economic growth. Therefore, the preference for green finance may not be desirable in these cities. Moreover, governments must account for the different industrial structures, resources, economic sizes, and financial development bases of their designated green finance reform pilot zones, reflecting each unique context. As such, the designations and functional positioning of each pilot zone may require tailoring to best suit local conditions, supply capacity, and demand scales accordingly.

One potential limitation of this study is that the sample data only reflects information up to 2019. Given that the first announcement of green financial reform and innovation pilot zones was only released in June 2017, this means that the analysis covered a relatively brief period. Additionally, the COVID-19 pandemic that emerged in 2020 required significant efforts to combat, which created further challenges in measuring the full impact of the pilot zones on the economy. To strengthen the accuracy of future research, longer-term observation of the impact of the pilot zones on economic growth, including more established cities, would be necessary. This would allow for a more comprehensive and precise analysis of the effects of the pilot zones on regional economic development, potentially mitigating the limitations of this study based on limited data.

While this paper has made a considerable effort to analyze the impact of green financial reform pilot zones on regional economic growth, there are several potential areas for future research that could enhance the study's strength. One such area is to conduct greater in-depth analysis into the different impacts of pilot zones on regions with varying industrial structures as the following study does not consider the pilot zones based on the industrial structures. This could involve further categorization of cities based on their specific industrial makeup, allowing for more detailed and precise insights into the relationship between green finance policies and economic performance as this will extend the following study further. Furthermore, since some of the pilot cities are also part of other policy experiments, such as low-carbon city pilots, it may be difficult to entirely eliminate any possible influences of these factors in our study throughout the robustness test stage. Therefore, it would be essential to exclude potential effects of unaccounted variables more thoroughly in future studies. As some of the cities included in our study are in embryonic stage and the green finance reform and innovation pilot zone is still undergoing exploration and promotion stages, and it may take more time to determine whether its potential to provide economic growth stimuli is limited. Thus, over time, the potential complexities and multidimensional impacts of green finance policies may further unfold, and this would provide more opportunities for research expansion.

## Author contribution statement

Cheng Hu: Conceived and designed the experiments; Performed the experiments; Analyzed and interpreted the data; Contributed reagents, materials, analysis tools or data; Wrote the paper.

Yan Wang: Contributed reagents, materials, analysis tools or data.

## Funding information

This work was supported by the [Young Talent Project Fund of Science and Technology Research Program of Hubei Provincial Education Department] (Q20223101).

## Data availability statement

The data that support the findings of this study are available on request from the corresponding author. The data are not publicly available due to privacy or ethical restrictions.

## Declaration of competing interest

The authors declare that they have no known competing financial interests or personal relationships that could have appeared to influence the work reported in this paper.
